# Host Molecules Regulating Neural Invasion of Zika Virus and Drug Repurposing Strategy

**DOI:** 10.3389/fmicb.2022.743147

**Published:** 2022-03-04

**Authors:** Li Yin Tan, Thamil Vaani Komarasamy, William James, Vinod R. M. T. Balasubramaniam

**Affiliations:** ^1^Infection and Immunity Research Strength, Jeffrey Cheah School of Medicine and Health Sciences, Monash University Malaysia, Bandar Sunway, Malaysia; ^2^Greenslopes Private Hospital, Greenslopes, QLD, Australia; ^3^Sir William Dunn School of Pathology, University of Oxford, Oxford, United Kingdom

**Keywords:** zika virus, blood-brain barrier, transcytosis, Trojan horse, inflammatory response, endoplasmic reticulum stress, autophagy, drug repurposing

## Abstract

Zika virus (ZIKV) is a mosquito-borne, single-stranded RNA virus belonging to the genus Flavivirus. Although ZIKV infection is usually known to exhibit mild clinical symptoms, intrauterine ZIKV infections have been associated with severe neurological manifestations, including microcephaly and Guillain Barre syndrome (GBS). Therefore, it is imperative to understand the mechanisms of ZIKV entry into the central nervous system (CNS) and its effect on brain cells. Several routes of neuro-invasion have been identified, among which blood–brain barrier (BBB) disruption is the commonest mode of access. The molecular receptors involved in viral entry remain unknown; with various proposed molecular ZIKV-host interactions including potential non-receptor mediated cellular entry. As ZIKV invade neuronal cells, they trigger neurotoxic mechanisms *via* cell-autonomous and non-cell autonomous pathways, resulting in neurogenesis dysfunction, viral replication, and cell death, all of which eventually lead to microcephaly. Together, our understanding of the biological mechanisms of ZIKV exposure would aid in the development of anti-ZIKV therapies targeting host cellular and/or viral components to combat ZIKV infection and its neurological manifestations. In this present work, we review the current understanding of ZIKV entry mechanisms into the CNS and its implications on the brain. We also highlight the status of the drug repurposing approach for the development of potential antiviral drugs against ZIKV.

## Introduction

Zika virus (ZIKV) is a mosquito-vectored flavivirus, consisting of three structural proteins [capsid (C), pre-membrane/membrane (prM/M), and envelope (E)], seven non-structural (NS) proteins (NS1, NS2A, NS2B, NS3, NS4A, NS4B, and NS5) and a single-stranded RNA genome of positive polarity (Yun and Lee, 2017). ZIKV has been detected in saliva, tears, urine, semen, brain, female genital tract, and testes (Morrison and Diamond, 2017). ZIKV along with the other members of the Flaviviridae family, including West Nile virus (WNV), dengue virus (DENV), hepatitis C virus (HCV), and Japanese encephalitis virus (JEV) possess significant neuroinvasive characteristics and are identified as neurotropic. In recent years, ZIKV received global attention due to the association with more severe neurological manifestations, such as Guillain-Barre syndrome (GBS) in adults (Oehler et al., 2014; Cao-Lormeau et al., 2016; Watrin et al., 2016) as well as microcephaly in infants (Brasil et al., 2016; Schuler-Faccini et al., 2016). Recent studies have demonstrated the neuro-invasiveness, tropism, and virulence of ZIKV (Shao et al., 2016; Costa et al., 2017a; Zhang et al., 2019). Owing to these severe complications, an extensive understanding of ZIKV neuroinvasion mechanisms and the host molecules involved is vital for therapy development.

## Receptors in Central Nervous System Targeted by ZIKV

The entry mechanism of ZIKV into human cells, particularly neural cells, remains poorly understood. Previous studies have demonstrated several ZIKV entry factors, including dendritic cell-specific intercellular adhesion molecule-3-grabbing non-integrin (DC-SIGN), AXL receptor tyrosine kinase (AXL), TYRO3 protein tyrosine kinase (TYRO3) and though to a lesser extent, T-cell immunoglobulin and mucin domain 1 (TIM-1), mediate entry of ZIKV into human dermal fibroblasts, epidermal keratinocytes, and immature dendritic cells (Hamel et al., 2015; Table 1). It is of note that ZIKV infection in primary dermal fibroblast was significantly decreased by RNA inhibitor and neutralizing antibody to AXL (Hamel et al., 2015). Therefore, the AXL receptor appears to play a vital role as the viral entry receptor. High expression of AXL receptor is seen within cells of the developing central nervous system (CNS), such as endothelial cells, microglial, astrocytes, and radial glial cells (Table 1; Nowakowski et al., 2016). A study found that the susceptibility of ZIKV to endothelial cells positively correlated with the cell surface levels of AXL (Liu et al., 2016). Belonging to the group of tyrosine kinase receptors TYRO3, AXL, and MER (TAM) family, AXL acts in apoptotic cells clearance and innate immunity modulation (Rothlin et al., 2007; Lemke and Rothlin, 2008). It is said that ZIKV is attached indirectly to the AXL receptor, mediated by the natural ligand of AXL, growth arrest–specific gene 6 (Gas6) based on the exposure of phosphatidylserine (PS) on viral envelope surface. Astrocytes and microglial cells appear to be the major ZIKV targets, having remained strongly expressed in the developing human cortex even as gestation progress (Meertens et al., 2017).

**Table 1:** Host cell entry receptors targeted by ZIKV (Lee et al., 2018; Lee and Shin, 2019).

Tissue sites	Cells	Entry receptors
Brain	Neural progenitor cells (NPCs)	AXL receptor tyrosine kinase (AXL), TLR3
	Astrocytes	AXL
	Microglial cells	AXL
Retina	Retinal pericytes	AXL, TYRO3
	Retinal microvascular endothelial cells	AXL, TYRO3
Blood	Dendritic cells	Dendritic cell-specific intercellular adhesion molecule-3-grabbing non-integrin (DC-SIGN)
	Monocytes (CD14+, CD16+)	Unknown
Placenta	Hofbauer cells	AXL, TIM-1, and TYRO-3
	Trophoblasts	AXL, TIM-1, and TYRO-3
	Endothelial cells	AXL, TIM-1, and TYRO-3
Kidney	Renal mesangial cell	Unknown
	Glomerular podocytes	Unknown
	Renal glomerular endothelial cell	Unknown
Testis	Spermatozoa	TYRO3
	Sertoli cells	AXL
Skin	Epidermal keratinocytes	AXL, TIM-1, and TYRO-3
	Dermal fibroblasts	AXL, TIM-1, and TYRO-3

However, recent findings demonstrated that ZIKV still showed effective invasion, infection, and replication in AXL-depleted cerebral organoids (Wells et al., 2016), neural progenitor cells (NPCs; Wells et al., 2016), and murine models (Wang et al., 2017b; Li et al., 2017c), suggesting that AXL may not serve as the exclusive receptor involved and that its dependency for viral entry may be cell-type or model specific. A further study reported the role of AXL in antagonizing ZIKV-induced activation of type I interferon (IFN) signaling, which facilitates ZIKV infection in astrocytes, instead of being a ZIKV entry receptor (Chen et al., 2018). While active investigations are still required on ZIKV target receptors, other receptor-independent mechanisms should be considered. Exosomes, secreted by most cell types, transport proteins, lipids, and nucleic acids between cells through systemic circulation or paracrine transmission, thereby exerting functional responses in target cells (Ramakrishnaiah et al., 2013). Several studies have shown exosome-mediated intercellular transmission of viruses (Narayanan et al., 2013; Ramakrishnaiah et al., 2013; Zhu et al., 2015). Tunneling nanotubes that connect a number of cell types, including immune and neuronal cells, are also shown to promote viral spread (Sowinski et al., 2008).

Tan et al. (2019) studied the mechanism underlying ZIKV infection in Vero, Huh-7, and induced pluripotent stem cell (iPSC)-derived human NPCs (hNPCs) using cell surface carbohydrates, sialic acid, and being the attachment receptor for several viruses. Although there was no direct involvement with ZIKV attachment, findings suggested that sialic acid could be an important mediator in the internalization of the ZIKV-receptor complex and its depletion significantly reduced infection in NPCs (Tan et al., 2019). Nevertheless, the underlying mechanism remains unclear and requires further research (Tan et al., 2019). Other factors, such as neural cell adhesion molecule 1 (NCAM1) and integrin αVβ5 have been postulated to act as cell-type-specific receptors for ZIKV entry (Srivastava et al., 2020; Wang et al., 2020).

## The Mechanisms of ZIKV Crossing the BBB

The blood–brain barrier (BBB) comprises endothelial cells strongly adhered *via* tight junction proteins (TJP), which are associated with pericytes, astrocytes, and microglia (Abbott et al., 2010; Mustafa et al., 2019). The tight junctions (TJ) complexes of transmembrane proteins composed of structural proteins claudins and occludins found on plasma membranes of adjacent brain endothelial cells maintain the integrity of BBB. Several routes of invasion into the CNS have been demonstrated by neurotropic viruses: (Yun and Lee, 2017) transcellular (through cells) transport within endothelial cells through infection or transcytosis, and subsequent release of the virus into the CNS, (Morrison and Diamond, 2017) infected peripheral immune cells such as monocytes enter the CNS through Trojan horse strategy, (Oehler et al., 2014) paracellular (between cells) entry of virus following disruption of the BBB, and destabilization of TJ, (Cao-Lormeau et al., 2016) retrograde axonal transport of virus through peripheral nerves into the CNS, and (Watrin et al., 2016) blood-to-cerebral-spinal-fluid (CSF) translocation (Ayala-Nunez and Gaudin, 2020; Hsieh and St John, 2020; Chen and Li, 2021). Among these, BBB dysfunction is the most common neuroinvasion route for flavivirus due to indirect effects of systemic inflammatory cytokines such as tumor necrosis factor (TNF) and IFN or direct attachment to claudins (Neal, 2014).

**Figure 1:**
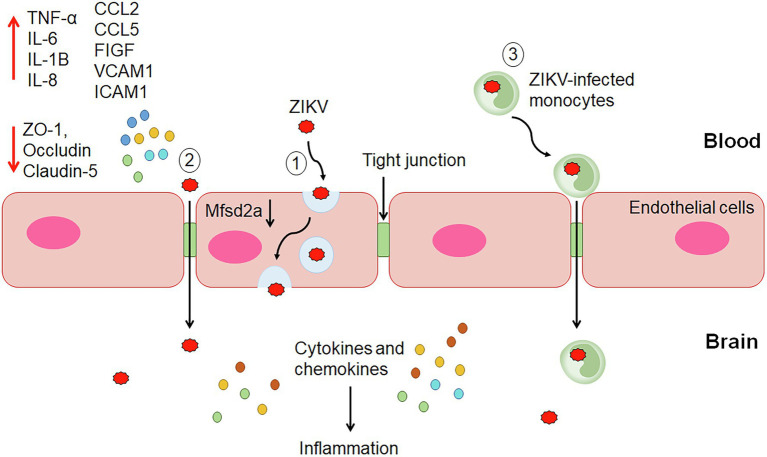
The proposed mechanisms of ZIKV invasion into the central nervous system (CNS). (1) Transcellular transport within endothelial cells of the BBB through infection or transcytosis mediated by ZIKV-induced degradation of Mfsd2a. (2) Paracellular trafficking of ZIKV across the blood–brain barrier (BBB) occurs through the upregulation of proinflammatory cytokines, chemokines, adhesion molecules and growth factors, and downregulation of tight junction proteins leading to alteration of the endothelial barrier integrity and permeability. (3) ZIKV-infected monocytes cross the BBB via the Trojan horse strategy. Once reach the CNS, ZIKV infects the brain cells, including astrocytes and microglial cells producing cytokines and chemokines leading to inflammation.

It was demonstrated that ZIKV can infect and efficiently replicate in the human brain microvascular endothelial cells (HBMECs) and iPSC-derived BBB models without significantly altering the endothelial barrier integrity and permeability *in vitro* (Mladinich et al., 2017; Papa et al., 2017; Alimonti et al., 2018). Despite the efficient viral replication, the endothelium displayed tight BBB and its architecture was not extensively perturbed. However, actin cytoskeleton rearrangement was observed upon infection, suggesting possible changes in the morphology of the endothelium (Cle et al., 2020). These findings suggest that endothelial leakage and BBB disruption may not be essential for ZIKV to reach the brain, and ZIKV might utilize other mechanisms to invade the CNS (Figure 1).

Several *in vitro* studies have demonstrated ZIKV infection and release from both apical and basolateral surfaces of brain endothelial cells without compromising the barrier permeability and integrity (Mladinich et al., 2017; Papa et al., 2017; Alimonti et al., 2018). ZIKV RNA was detected in the lower chamber of the transwell system despite maintaining the endothelial integrity. These studies support that ZIKV may cross the BBB through transcytosis, basolateral virus release, or paracytosis (Figure 1). A charge-based mechanism occurs whereby positively charged ZIKV particles foster attraction with the negatively charged membrane of brain endothelial cells for transcytosis (Nakayama et al., 2021). The secretion of type I and III IFNs and inflammatory cytokines did not affect microvascular endothelial cells (MECs) permeability but contributed to transinfection (Papa et al., 2017). These findings suggest potential drugs that block ZIKV replication or transcytosis can effectively prevent virus extravasation through MEC monolayer. Treatment with Chloroquine (inhibitor of cellular infection) and BFA (exocytosis inhibitor), inhibited virus RNA release and extravasation in a transwell system, indicating active virus replication is required for ZIKV to cross the BBB. On the other hand, treatment with Nystatin (inhibitor of caveolae-mediated transcytosis) impaired extravasation of ZIKV through HBMECs without affecting virus replication, suggesting utilization of transcytosis and/or basolateral release pathways by ZIKV to cross the BBB after infection and activation of endothelial cells (Papa et al., 2017).

The ZIKV transcytosis may be mediated by the major facilitator superfamily domain-containing protein 2 (Mfsd2a; Figure 1). Mfsd2a is selectively expressed on the CNS endothelial cells, and its genetic ablation resulted in increased transcytosis and leaky BBB (Ben-Zvi et al., 2014). It plays a critical role in transporting docosahexaenoic acid (DHA) into CNS (Chen and Li, 2021). The lipids transported by Mfsd2a mediate inhibition of caveolae vesicle formation in CNS endothelial cells and suppress transcytosis through which it maintains the integrity of the BBB. The deficiency of Msfd2a reduced the levels of DHA in the brain, which was accompanied by loss of neuronal cells, cognitive deficits, and microcephaly (Nguyen et al., 2014). ZIKV envelope (E) protein was found to interact with Mfsd2a. ZIKV E promotes polyubiquitination of Mfsd2a and mediates its proteasome-dependent degradation. ZIKV inhibited the levels of Mfsd2a in hBMECs and in neonatal mouse brains. In addition, ZIKV caused a reduction in the Mfsd2a-mediated DHA uptake and supplementation with DHA rescued ZIKV-indued abnormal brain development in mice (Zhou et al., 2019).

A study by Ayala-Nunez et al. (2019) proposed that ZIKV may use the Trojan horse strategy to cross the BBB by infecting the monocytes (Figure 1). The study found ZIKV-infected monocytes in the brain slices from the fetus with microcephaly. It was further demonstrated that ZIKV productively infected human primary monocytes and promoted viral dissemination to cerebral organoids. ZIKV manipulates the adhesive properties of monocytes, particularly CD14 and CD16 monocytes by increasing its expression of surface adhesion molecules (integrins, ICAM3, PECAM1, IQGAP1, catenin, myosins, actinin, KIF5B, vinculin, talin, and filamin A and B). This enables the ZIKV-infected monocytes to have a greater attachment to the blood vessel wall and transmigrate across the endothelium to infect neural cells (Ayala-Nunez et al., 2019). A study showed that HBMECs allowed transmigration of ZIKV-infected human monocyte THP-1 in a transwell system and subsequently infected the astrocytes in the basolateral compartment. As astrocytes play an important role in the maintenance of BBB through direct interaction with endothelial cells in the brains, ZIKV infection of these cells after crossing the endothelial layer may lead to inflammation and alteration of the barrier. In this context, modulation of inflammatory molecules, such as, C-C motif ligand-5 (CCL5/RANTES), C-X-C motif chemokine ligand 10 (CXCL10), and IFN-β was observed in astrocytes (Bramley et al., 2017; Cle et al., 2020). ZIKV infection of monocytes was also observed in macaques, and the infected cells were recruited to tissues, resulting in persistent viral infection (O’Connor et al., 2018). Hence, ZIKV-infected monocytes could represent a carrier for the Trojan horse strategy to invade the CNS.

The Trojan horse transmigration of ZIKV-infected monocytes across BBB depends on two receptors, chemokine receptor 7 (CCR7) and receptor for advanced glycation end (RAGE) expressed on ZIKV-infected monocytes. In a state of inflammation, the natural ligand of CCR7, chemokine ligand 19 (CCL19), and the danger-associated molecular pattern (DAMP) molecule, nuclear high mobility group box 1 (HMGB1) are upregulated. This receptor-ligand interaction induces dysregulation of the endothelium layer associated with disorganization of cadherins and actin fibers, thereby increasing the membrane’s permissiveness (de Carvalho et al., 2019). The process is mediated by the upregulation of C-X-C motif chemokine 12 (CXCL12) in monocyte during ZIKV infection, a key regulator of lymphocytes shifts across BBB into CNS parenchyma as it interacts with C-X-C chemokine receptor type 4 (CXCR4). The accompanying lymphocytes-induced inflammation in brain parenchyma further triggers subsequent downstream activation pathways resulting in increased BBB damage (Panganiban et al., 2020).

The paracellular pathway involving proteasomal degradation mechanism has been proposed as one of the pathways for BBB penetration (Figure 1). A study by Shao et al. showed that ZIKV infection induced altered vasculature and a leaky BBB in developing mouse brain (Shao et al., 2016). An *in vitro* study using transfected HBMECs (THBMEC) infected with ZIKV demonstrated vascular leakage enhancement by ZIKV through disruption in the cytoskeleton and tight junctional proteins arrangement. This occurs *via* the upregulation of genes that are involved in the production of proinflammatory cytokines, such as interleukin-6 (IL-6), TNF-α, cell adhesion molecules (CAMs), and growth factors (Ismail et al., 2018), which activates the RhoA/Rho-associated coiled-coil containing protein kinase (ROCK)/phosphorylated myosin light chain (pMLC) signaling effectors, thus intensifying stress fibers production (Chen and Li, 2021). Additionally, matrix metalloproteinases (MMP-2/MMP-9) and the proteasome are stimulated by the RhoA mechanism in the breakdown of TJP (Chen and Li, 2021). Downregulation of TJP zonula occludens-1 (ZO-1), occludin, and Claudin-5 by ZIKV (Leda et al., 2019), affecting their ability for transient phosphorylation and dephosphorylation (Siddiqui et al., 2015), was linked to an increase in trans endothelial permeability and BBB penetration (Argaw et al., 2009). The ZIKV-induced BBB disruption is likely to occur at later stages of the disease (Papa et al., 2017; Leda et al., 2019).

Zika virus infection of HBMECs and *in vitro* BBB model enhanced expression of type I and III IFNs and induced a significant increase in the release of cytokines [interleukin-1β (IL-1β), interleukin-8 (IL-8), IL-6, and TNF-α], chemokines [C-C motif ligand-2 (CCL2/MCP-1) and CCL5], and CAMs [vascular cell adhesion molecule 1 (VCAM1) and intercellular adhesion molecule 1 (ICAM1)]. ZIKV infection of pericytes showed upregulation of chemokines (CCL5 and CXCL10), cytokines [IL-6, IL-8, and interleukin-15 (IL-15)], as well as Toll-like receptor 3 (TLR3; Cle et al., 2020). The modulation of BBB proteins induced by ZIKV infection of endothelial cells and pericytes may trigger recruitment and docking of immune cells to the BBB and potentially lead to immune cell CNS infiltration and neuroinflammation (Papa et al., 2017; Cle et al., 2020). Increased levels of CAM were also observed in ZIKV-infected mouse models as well as in plasma from patients. In a mouse model, BBB permeability was not observed during the early time points, but subtle BBB alteration was detected at later time points, which could be associated with an inflammatory response triggered by viral replication (Papa et al., 2017). A study by Bramley et al. (2017) demonstrated that pre-treatment of a three-dimensional (3D) model of microvascular endothelial cells with TNF-α enhanced virus replication and disorganization of the junctional network, suggesting the potential role of inflammatory response in BBB disruption *in vivo*.

As a matter of interest, cerebral organoid designs hold promising means in studying the 3D morphological and molecular characterization in ZIKV-induced microcephaly as it is able to recapitulate the human brain environment (Antonucci and Gehrke, 2019). However, this system has limited vascularization required for the prolonged culture period, presenting difficulty in discerning the necrotic core due to ZIKV or suboptimal preparation (Antonucci and Gehrke, 2019; Chen et al., 2019). The lack of BBB affects the ability to emulate the natural neuroimmune response toward ZIKV by infiltrating peripheral immune cells across the BBB (Antonucci and Gehrke, 2019). Therefore, co-culturing or other bioengineering techniques would improve the brain organoid model and provide a clear picture of the relationship between the CNS and peripheral circulatory system (Chen et al., 2019).

On the other hand, ZIKV infection and dissemination along peripheral nerves lack evidence and may not be the primary mechanism; however, it cannot be fully disregarded. Contrary to the BBB endothelial cells, the choroid plexus endothelial cells have fenestrated blood capillaries and are leaky, thereby could provide a pathway for the virus to spread out of the blood and enter into the choroid plexus cisternae. ZIKV can infect the pericytes around the fenestrated capillaries, forming a local amplification site and subsequently transcytosed across the blood-CSF layers (Kim et al., 2020).

## The Implications of ZIKV Infection on the Brain

Similar to primary microcephaly, ZIKV-related microcephaly was reported to be the neurodevelopmental disruption during the first trimester when cortical neurogenesis is most active (Johansson et al., 2016). ZIKV causes microcephaly by affecting the NPCs through cell-autonomous and non-cell autonomous pathways.

Cell cycle perturbation by ZIKV, in particular the S-phase restriction, provides a favorable cellular environment for ZIKV replication as well as impairs the growth of hNPCs (Hammack et al., 2019). ZIKV causes host DNA breaks, activating the ataxia telangiectasia mutated (ATM)/Chk2 signaling cascade and inhibiting ataxia telangiectasia and rad3+ related (ATR)/Chk1 signaling cascade (Hammack et al., 2019). DNA damage response (DDR) is subsequently activated where DNA repair proteins H2A.X and 53BP1 are phosphorylated and cell cycle regulators proteins cell division cycle 25 A (CDC25A), cyclin A and cyclin E are degraded, resulting in G1/S transition arrest (Hammack et al., 2019). G0/G1 transition arrest was not shown to enhance ZIKV replication, implying a possible need for host DNA replication or damage repair factors for its replication (Hammack et al., 2019). Another *in vitro* study found that ZIKV envelope proteins restrict G2/M progression and induce apoptosis in neuroendocrine PC12 cells through upregulation of tumor suppressor protein, p53 and cyclin-dependent kinase (CDK) inhibitor (CDKi), p21^Cip1/Waf1^ leading to downregulation of G2/M phase regulator, cyclin B1, and augmentation of the proapoptotic pathway with the increased B-cell lymphoma protein 2 (Bcl-2)-associated X (Bax)/Bcl-2 ratio (Liu et al., 2018). In this context, the intrinsic cell death signaling mechanism is triggered by the activation of caspase-9 and caspase-3 (Liu et al., 2018). The attenuation of neural cell proliferation in the ventricular, subventricular, and intermediate zone of the fetal brain causes a significant decrease in intermediate progenitor cells, lateral ventricles size, and cortical surface area (Wu et al., 2016). Additionally, defective cell division due to ZIKV may precede the apoptotic program of neural stem cells; a cellular mechanism used to prevent genomic instability (Vitale et al., 2011; Souza et al., 2016). A number of mitotic dysfunctions were reported, such as extra centrosomes, multipolar spindles, partial segregation of spindle pole, chromosomal aneuploidy, and micronuclei production (Souza et al., 2016).

**Figure 2:**
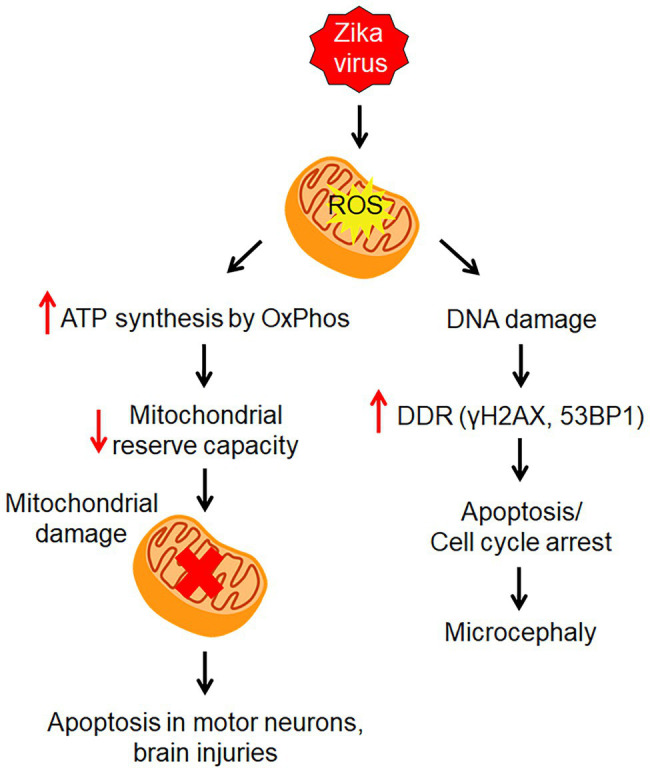
Zika virus-induced mitochondrial stress. ZIKV infection causes mitochondrial stress by altering its structure and metabolism to supply energy for viral replication through oxidative phosphorylation (OxPhos) pathway. This leads to an increase in reactive oxygen species (ROS) which causes DNA damage. Then the DNA damage response (DDR) is induced to activate repair pathways to monitor DNA damage.

Recent findings revealed that ZIKV causes mitochondrial stress in human derived-iPSC astrocytes with alteration to both its structure and metabolism to supply the energy demand for viral replication through oxidative phosphorylation (OxPhos) pathway of ATP production (Ledur et al., 2020; Figure 2). Mitochondria being the putative avenue for reactive oxygen species (ROS) production were observed to have an increased level of oxidative stress in infected astrocytes, contributing to the DNA breaks and activation of DDR (Ledur et al., 2020; Figure 2). DDR plays an important role in inducing cell cycle arrest apoptosis (Devhare et al., 2017). The activation of DDR triggered phosphorylation of Histone H2AX (γH2AX) followed by assembly of 53BP1, both of which interact with other DNA repair proteins at the foci of DNA double-stranded break (Ledur et al., 2020; Figure 2). DDR plays an important role in inducing cell cycle arrest apoptosis (Devhare et al., 2017). Intermediate filaments, glial fibrillary acidic protein (GFAP), and Vimentin were elevated in infected astrocytes *in vitro* and *in vivo*, corroborating with reactive astrogliosis induced by ZIKV (Ledur et al., 2020). That said, mitochondrial dysfunction has been known to be associated with various neurological disorders, including neurodegenerative diseases, cerebral hypoxia, cerebral ischemia, and other brain injuries (Lopez-Domenech et al., 2016; Alimonti et al., 2018; Ludwig et al., 2018; Zhao et al., 2019). In the context of astrocytes, mitochondrial damage has been shown to cause apoptosis in motor neurons, and axon destruction in GBS (Madigan et al., 2017).

**Figure 3:**
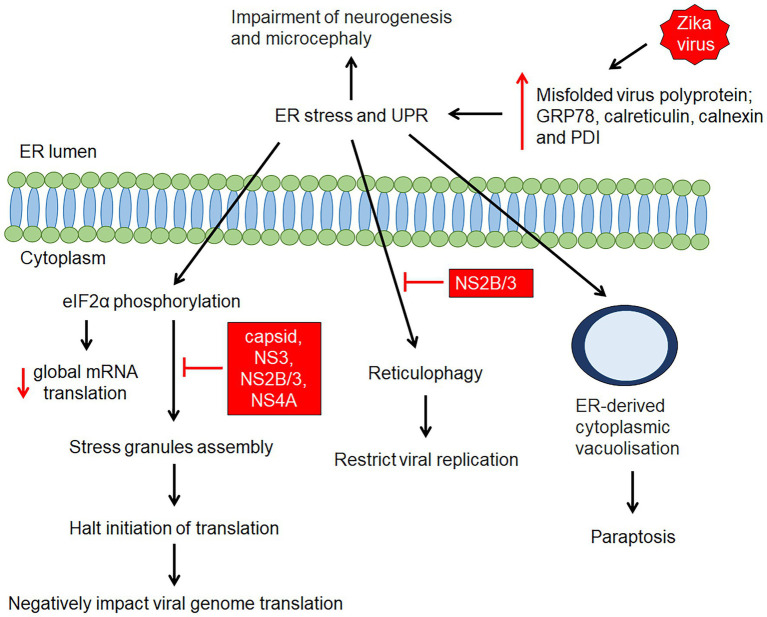
ZIKV-induced endoplasmic reticulum (ER) stress and unfolded protein response. ZIKV infection causes structural changes of the ER as a result of accumulation of misfolded/unfolded ZIKV proteins and remodelling of the ER structure for viral RNA replication. The accumulation of misfolded/unfolded ZIKV proteins and the increase in ER protein-folding capacity triggers ER stress and activates the unfolded protein response (UPR), resulting in elevation of GRP78, calnexin, calreticulin, and protein disulfide isomerase (PDI). This is followed by initiation of several mechanisms such as global protein translation, stress granule assembly, reticulophagy and cytoplasmic vacuolisation. Zika virus proteins (capsid, NS3, NS2B/3 and NS4A proteins) has shown to, suppress SGs assembly, while NS2B/3 has shown to inhibit reticulophagy which facilitate continuous viral replication.

Zika virus is capable of modulating the host endoplasmic reticulum (ER) structure for its replicative benefits (Ropidi et al., 2020; Figure 3). The viral NS4A exploits host reticulon 3.1A to promote ER membrane curvature for viral entry into ER, allowing replication to take place (Ropidi et al., 2020). The budded ZIKV RNA, along with other ZIKV proteins such as NS2B-NS3 and unprocessed C-prM-Env complexes are then assembled and cleaved in adjacent ER by ZIKV NS2A protein and NS2B-NS3 protease, respectively thereby producing new ZIKV virions (Ropidi et al., 2020). However, in the process of ZIKV infection, excessive formation of misfolded proteins overpowers the ER protein-folding capacity, causing ER stress and activation of unfolded protein response (UPR), which ultimately lead to apoptosis (Ropidi et al., 2020; Figure 3). *In vitro* and *in vivo* studies have shown an increase in ER stress proteins as well as expression of key molecules of the UPR, such as glucose regulatory protein 78 (GRP78), calreticulin, calnexin, and protein disulfide isomerase (PDI) in infected neural cells (Ropidi et al., 2020), resulting in neurogenesis inhibition and microcephaly (Gladwyn-Ng et al., 2018; Figure 3). In parallel, ZIKV-induced ER stress represses stress granules (SG) assembly, which functions to arrest global translation *via* eIF2α phosphorylation and SG proteins exploitation (Ropidi et al., 2020; Figure 3). Reticulophagy, a compensatory host innate defense response to engulf viral protein and damaged ER for lysosomal degradation, is inhibited by ZIKV NS2B-NS3 protease (NS2B/3; Ropidi et al., 2020; Figure 3). Besides intrinsic and extrinsic apoptotic cell death, the prolonged ER stress renders paraptosis-like death through extensive cytoplasmic vacuolization in ZIKV-infected cells (Ropidi et al., 2020; Figure 3).

Modulation of the host autophagy mechanism has been demonstrated by flaviviruses, such as DENV (Heaton and Randall, 2010), HCV (Sir et al., 2012), and ZIKV in skin fibroblast (Hamel et al., 2015) to promote viral replication. Recent findings showed that ZIKV induced the autophagosome-specific marker, cytosolic microtubule-associated light chain 3 (LC3), indicating autophagosomes are proviral and provide the avenue for ZIKV replication (Liang et al., 2016). ZIKV NS4A and NS4B have shown to inhibit the AKT-mammalian target of rapamycin (mTOR) signaling, resulting in reduced neurogenesis and increased autophagy in human fetal neural stem cells, therefore elevating viral replication (Liang et al., 2016). Although the precise molecular mechanism by ZIKV on autophagosomes is still elusive, various pathways, such as deregulating the host antiviral innate immunity (Ke and Chen, 2011), upregulating viral RNA translation (Dreux et al., 2009), and modulating host lipid metabolism for viral replication (Heaton and Randall, 2010) have been proposed and would benefit from further exploration.

From the non-cell autonomous pathway aspect, a recent study demonstrated that ZIKV induced neuronal apoptosis on adjacent healthy neurons while maintaining its replication in the infected neurons, potentially *via* ZIKV’s upregulation of pre-mRNA-processing-splicing factor 8 (Pprf8), an anti-apoptotic factor demonstrated in Picornavirus-infected neurons (Olmo et al., 2017). ZIKV-infected neurons undergo neuroinflammation process, secreting TNF-α, IL-1β, and glutamate (Olmo et al., 2017). These neurotoxic factors activate GluN2B-containing N-methyl-d-aspartate receptor (NMDAR), potentiating Ca2+ influx into the cell, which promotes excitotoxicity and eventually cell death (Olmo et al., 2017).

Cortical development is a highly complex process requiring tightly regulated and finely tuned sequential machinery. Hence, as vascular development is pivotal for organogenesis, the impairment of neurogenesis is demonstrated to be in part, due to vascular abnormalities (Garcez et al., 2018). Defective vasculature and decreased neuronal proliferation were concurrently apparent in the ZIKV-infected mice brain models, particularly in the ventricular zone, indicating the presence of a causal relationship between them (Garcez et al., 2018). These findings are further supported by the significant elevation in anti-angiogenic proteins, Ang1, Ang3, as well as endostatin and pigment epithelium-derived factor (PEDF), albeit to a smaller degree from proteomic analysis (Garcez et al., 2018).

## Drug-Repurposing Strategy for the Treatment of ZIKV

Since the 2015 ZIKV outbreak and its association with congenital abnormalities, tremendous progress has been made in vaccine and antiviral research. More than 25 ZIKV vaccine candidates have been evaluated in nonclinical development and at least 12 are in clinical evaluations. These candidates include DNA, mRNA, viral-vectored, inactivated, and live attenuated vaccines (Barrett, 2018; Poland et al., 2019; Lee et al., 2021). Some of these candidates have demonstrated the ability to prevent viral transmission during pregnancy, as well as fetal malformations and demise in animal models (Richner et al., 2017; Shan et al., 2017; Li et al., 2018). Multiple candidates have been shown to be safe, well-tolerated and immunogenic in humans, with two candidates (VRC5288 and mRNA-1893) having advanced into phase 2 clinical trials (Barrett, 2018; Lee et al., 2021). In addition, therapeutic vaccination for ZIKV has been extensively explored as an alternative to vaccines. A total of 461 monoclonal antibodies (mAbs) that bind to E proteins have been identified, with 70 of them displaying moderate to high neutralizing activities (Wang et al., 2017c). Notably, administration of convalescent serum from a ZIKV-infected patient not only inhibited ZIKV replication but also prevented microcephaly in a mouse model (Wang et al., 2017a). While treatment with a human mAb, ZIKV-117 reduced vertical transmission and improved fetal outcome (Sapparapu et al., 2016). Another study showed that a cocktail of three neutralizing mAbs targeting different domains of the ZIKV E protein completely prevented viremia in non-human primates (NHPs; Magnani et al., 2017).

On the other hand, several approaches have been employed to identify drugs against ZIKV infection. The current search for ZIKV antivirals can be classified based on their mode of action, such as (i) host-directed antivirals, which focus on modulating host cellular processes used for viral life cycle or (ii) direct-acting antivirals, which target viral components. In order to expedite the development of effective antivirals against ZIKV, much focus has been given to drug repurposing or drug re-profiling (Table 2). Recommended therapies for ZIKV infection would require the ability to cross the placenta and BBB, have anti-ZIKV functionality primarily on fetal neural cells and be safe to use during pregnancy.

**Table 2:** Potential anti-ZIKV drugs focusing on studies done on neuronal cells.

Drug	Function	FDA approval/pregnancy	BBB permeation	Placental barrier permeation	Reference
MYD1	Decoy AXL receptor	-	No data yet	No data yet	Meertens et al. (2017)
R428	AXL kinase inhibitor	-	No data yet	No data yet	Meertens et al. (2017)
Nanchangmycin	Antibacterial & insecticidae	-	No data yet	No data yet	Rausch et al. (2017)
25-hydroxycholesterol	Endogenous oxysterol	-/Safe	Yes	No data yet	Li et al. (2017b)
Chloroquine	Antimalarial, anti-inflammatory and antiviral	Approved/C	Yes	Yes	Delvecchio et al. (2016)
Mefloquine	Antimalarial	Approved/B	Yes	Yes	Barrows et al. (2016)
*N*-(4-hydroxyphenyl)-retinamide/Fenretinide/4-HPR	Anticancer	-/Safe (past studies showed minimal & reversible side effect at high dosage only)	Yes	Yes	Formelli et al. (1998); Puduvalli et al. (2004); Pitts et al. (2017)
Emricasan/IDN-6556/PF-03491390	pan-caspase inhibitor	Approved/−	No data yet	No data yet	Xu et al. (2016); Munjal et al. (2017)
PHA-690509	cyclin-dependent kinase (CDK) inhibitor	Approved/D	No data yet	No data yet	Xu et al. (2016)
Seliciclib	CDK inhibitor used as anticancer	Approved/C	Yes but 30% less than plasma level	No data yet	Xu et al. (2016); Noonan et al. (2019)
RGB-286147	CDK inhibitor	-	No data yet	No data yet	Xu et al. (2016)
Bithionol	Antihelminthic; used to treat mouth & throat disorders; treat cerebral paragonimiasis	Approved/C	Yes	Yes	Leonardi et al. (2016)
NGI-1	oligosaccharyltransferase inhibitor	-	No data yet	No data yet	Puschnik et al., 2017
6-methylmercaptopurine riboside/6MMPr	Immunosuppressant, antiviral against HCV, bovine viral diarrhoea virus, yellow fever virus, dengue virus (DENV)-2, West Nile virus (WNV)	-	Lim et al. (2011) suggested poor CNS bioavailability but no data on BBB penetration	Yes, but limited diffusion across	Lim et al. (2011)
Ribavirin	Guanosine analog to treat influenza A and B, severe respiratory syncytial virus, Lassa fever virus and hepatitis C	Approved/X	No but using cyclodextrin as drug carrier significantly increase transport across	Equivocal, *In vitro* & *in vivo* studies have showed possible placental permeation	Jeulin et al. (2009); Kim et al. (2018); Karbanova et al. (2019)
Heparin	Anticoagulant	Approved/C	Yes	No	Ghezzi et al. (2017)
Memantine	NMDAR inhibitor used to treat Alzheimer’s disease	Approved/B	Yes	Yes	Costa et al. (2017); Victorino et al. (2017)
Azithromycin	Antibiotic	Approved/B	Yes	Yes	Jaruratanasirikul et al. (1996); Retallack et al. (2016)
IL-1 receptor antagonist/Kineret/Anakinra	Immunomodulator used to treat rheumatioid arthritis	Approved/B	Yes	Yes	Briggs et al. (2015); Cavalli and Dinarello, (2018); Lei et al. (2019)

### Host-Directed Antivirals

#### Attachment/Entry

MYD1, an engineered decoy AXL receptor, has a high-affinity binding to Gas6, preventing the interaction between ZIKV viral particles and the AXL receptor (Meertens et al., 2017). AXL kinase inhibitor R428 prevents phosphorylation of AXL, which activates the host innate immunity (Meertens et al., 2017). Both MYD1 and R428 inhibit ZIKV infection of human glial cells in a dose-dependent manner and are identified as potential antivirals through the AXL/Gas6 pathway inhibition (Meertens et al., 2017).

Nanchangmycin, an antibacterial and insecticidal polyether, potently inhibits the early entry stage in the ZIKV replication process and infection in primary cells of uterine, placenta, umbilical vein, BBB, and neuron-glial mixed midbrain at 0.1–0.4 μM with low cytopathic effects (Rausch et al., 2017).

25-hydroxycholesterol (25HC), an endogenous oxysterol formed *via* cholesterol oxidization, demonstrated antiviral activity by preventing ZIKV entry through internalization and fusion of ZIKV envelope with host endosomal membrane, not *via* ZIKV attachment (Li et al., 2017b). It decreased viral load and mortality in mice, inhibited ZIKV RNA shedding and symptoms in NHPs as well as protected developing human cortical organoids and embryonic mouse brain from ZIKV-associated neurological impairment and fetal microcephaly, respectively (Li et al., 2017b). 25HC displayed effectiveness in Vero cells at a concentration as low as 0.4 μM with no observable cytotoxicity at a concentration up to 10 μM. No adverse effects were seen on pregnant and neonatal mice at 50 mg/kg of 25HC (Li et al., 2017b).

#### Endosomal Fusion

Chloroquine, known for its antimalarial, anti-inflammatory, and antiviral activity, has been shown to protect Vero cells, HBMECs (*in vitro* BBB model), hNPCs, and mouse neurospheres from ZIKV infection (Delvecchio et al., 2016). It acts by increasing the endosomal pH, consequently preventing the fusion of ZIKV-host endosomal membrane, suppressing ZIKV-induced infections without exhibiting cytotoxicity (Delvecchio et al., 2016). Of note, chloroquine was said to be safe in pregnancy and able to penetrate the maternal-fetal placental barrier with a 4–30-fold higher concentration in the brain than in plasma (Delvecchio et al., 2016). These features are imperative in reducing the risk of infections and ZIKV-related microcephaly (Delvecchio et al., 2016).

Another antimalarial drug Mefloquine is proposed to work by preventing autophagy and interrupting the cellular lysosomal pH (Barrows et al., 2016). At a concentration of 10 μM, it was able to inhibit ZIKV infection to cervical HeLa cells, placental JEG3 cells, and primary human amnion epithelial cells (Barrows et al., 2016). Indeed, while further work is warranted to validate the beneficial anti-ZIKV effects of Mefloquine, the current data show promising anti-ZIKV effects, well-tolerated in pregnancy, as well as ability to cross to the placenta and the BBB (Barrows et al., 2016).

#### Translation/Transcription

*N*-(4-hydroxyphenyl)-retinamide (4-HPR or Fenretinide), known for its anticancer properties, is found to also exhibit anti-ZIKV activity, presumably facilitated by a host factor, in significantly reducing ZIKV RNA production without effect on viral polymerase or membrane-related replication complexes (Pitts et al., 2017). 4-HPR was shown to inhibit ZIKV in multiple mammalian cell lines culture as well as eliminate ZIKV viremia and brain viral load *in vivo* (Pitts et al., 2017).

#### Replication

Emricasan, a pan-caspase inhibitor (IDN-6556/PF-03491390), and PHA-690509, a CDKi, are able to suppress the caspase-3 pathway as well as improve hNPCs and astrocyte viability (Xu et al., 2016). Their effects are amplified even more when used in combination (Xu et al., 2016). Notably, the host cellular cyclin-dependent kinase (CDK) may serve as a valuable anti-ZIKV target given the close interconnection between CDKi, cell cycle regulation, and ZIKV proliferation (Xu et al., 2016). PHA-690509 alone is reported to partially protect hNPC against proliferation reduction and ZIKV replication (Xu et al., 2016). This inhibition effect on ZIKV production is corroborated by the identification of other CDKi, Seliciclib, and RGB-286147 with submicromolar half-maximal inhibitory concentration (IC_50_) at 24 and 27 nM, respectively (Xu et al., 2016). Bithionol, on the other hand, also blocks ZIKV-induced host caspases, possibly caspase-1, −3, −6, −7, and − 9, in Vero cells and human astrocytes (Leonardi et al., 2016). It is regarded highly as a potential therapy due to its ability to penetrate the placental and BBB and has been reported to be well-tolerated (Leonardi et al., 2016).

#### ER-Targeting Drugs

In various cell types, including hNPC, NGI-1 targets the ER-membrane multiprotein complex, and oligosaccharyltransferase (OST) complex, which is responsible for the catalysis of N-linked glycosylation of new proteins, by inhibiting viral RNA production and replication but independent of OST complex’s catalytic activity (Puschnik et al., 2017).

#### Nucleoside Biosynthesis

Azathioprine-derived thiopurine nucleoside analog, 6-methymercaptopurine riboside (6MMPr) was tested on epithelial and human neuronal cells, revealing efficient, dose-dependent inhibition of ZIKV infection, and lower cytotoxicity for neuronal cells (de Carvalho et al., 2017). It robustly blocks the *de novo* production pathway of purine, resulting in a smaller pool of nucleotides to be used for viral replication (de Carvalho et al., 2017). 6MMPr is reported to be well-tolerated during pregnancy and able to cross the placental barrier (de Carvalho et al., 2017). However, diffusion into the fetal vascular system did not reach a significant level (de Carvalho et al., 2017).

Ribavirin is a guanosine analog approved to treat influenza A and B, severe respiratory syncytial virus, Lassa fever virus, and hepatitis C (Kim et al., 2018). Similar to Favipiravir, Ribavirin showed a robust antiviral effect against ZIKV infection in hNPCs, as seen by the substantial dose-dependent decrease in mRNA expression of ZIKV E and NS5, with the greatest reduction at the 25 μg/ml (Kim et al., 2018). The study further demonstrated the extension of anti-ZIKV activity in human dermal fibroblasts, human lung adenocarcinoma cells, and Vero cells (Kim et al., 2018).

#### Cytopathic Effects Inhibition

Heparin, an anticoagulant, exhibited anti-apoptotic activity in hNPCs, potentially through inactivation of caspase-3 (Ghezzi et al., 2017). Its ultra-low molecular weight form is shown to infiltrate the BBB (Ghezzi et al., 2017). Unexpectedly, heparin only has a modest effect in curbing ZIKV infection and replication in hNPCs (Ghezzi et al., 2017). While heparin has no absolute contraindications during pregnancy, its inability to pass the placental barrier would necessitate a tailored drug delivery platform (Ghezzi et al., 2017).

Given that ZIKV can induce inflammation in infected neuronal cells and subsequent glutamate release to promote neurodegeneration of adjacent cells, Memantine which is a non-competitive NMDAR inhibitor used as Alzheimer’s disease therapy can inactivate NMDAR which are the primary ionotropic glutamate receptors in the brain, preventing high calcium influx and neurotoxicity at dosages 1, 10, and 30 μM (Costa et al., 2017b). The selective antagonism of Memantine toward overstimulated receptors presents lower cytotoxicity than other NMDAR antagonists and is thus classified as pregnancy class B by the US Food and Drug Administration (FDA; Costa et al., 2017b). Both *in vitro* and *in vivo* studies here demonstrated neuroprotective effects of memantine, Dizocilpine/MK-801, agmatine sulfate, and ifenprodil in ameliorating ZIKV-induced apoptosis without impacting the viral replication process (Costa et al., 2017b). Memantine was reported to be able to prevent microgliosis and overall brain injury, particularly in the cortical, striatal, and hippocampal regions (Costa et al., 2017b).

#### Unknown Mechanism

Antibiotic of macrolide-type Azithromycin, which is used to treat respiratory or sexually transmitted diseases, was reported to be able to cross the placental barrier and reach the fetal tissue, with concentrations of ~2.8 μM in placenta and 4–21 μM in the fetus (Retallack et al., 2016). Azithromycin was observed to decrease ZIKV infection, proliferation and cellular apoptosis in glial cells, and astrocytes (Retallack et al., 2016).

#### Placenta

As interleukin-1 (IL-1) receptors are expressed in microglial cells, IL-1 receptor antagonist (IRA) reduces fetal neuroinflammation by inhibiting fetal microglial activation (Lei et al., 2019). IRA indirectly prevents fetal neurocognitive abnormalities by preserving placental development despite existing ZIKV replication through its inhibition on ZIKV-induced placental proinflammatory cytokines IL-1β (Lei et al., 2019). This action reduces placental inflammation and increases trophoblast invasion and placental vascularity (Lei et al., 2019).

### Direct-Acting Antivirals

#### NS2B–NS3 Protease

Zika virus utilizes its encoded NS3 protein and NS2B cofactor for proteolytic cleavage of its polyprotein in the viral maturation process. One such compound efficacious in ZIKV inhibition through this mechanism is Niclosamide (NIC), an FDA-approved anthelmintic therapy. Consistent with previous *in vitro* findings using glioblastoma SNB-19 cells (Xu et al., 2016), NIC is able to inhibit ZIKV production, decrease proinflammatory proteins such as CXCL10 and leukemia inhibitor factor (LIF), partially restore altered neuronal differentiation profile, and inhibit apoptosis in human induced neural stem cell (hiNSC), with pre- and/or concomitant NIC treatment (Cairns et al., 2018). Using humanized *in vivo* embryo, NIC showed neuroprotective effects by partially restoring morphological change of the brain and differentiation profile in hiNSCs (Cairns et al., 2018). Together with niclosamide, temoporfin, a photosensitizer used for squamous cell carcinoma and nitazoxanide, an anthelmintic drug, share a similar mechanism in inhibiting NS2B-NS3 proteins interactions (Li et al., 2017a). Both cause inactivation of ZIKV protease and attenuation of ZIKV polyprotein precursor processing, thus abolishing ZIKV replication in human placental epithelial cells (HPECs), iPSC, and iPSC-derived hNPCs (Li et al., 2017a). All except temoporfin possess good safety profiles for use in pregnant patients (Li et al., 2017a).

#### NS5 RdRp

Sofosbuvir, which is a uridine nucleotide analog and anti-HCV drug, uses its 2′-F radical to form a covalent bond with amino acid residue Asn612 (Sacramento et al., 2017). The formation of this covalent bond impairs the subsequent hydrogen bonds formed between nucleotides and ZIKV RNA polymerase, therefore acting as a transcription terminator which directly blocks ZIKV RNA polymerase (ZVRP) in various cell models (Sacramento et al., 2017). Interestingly, sequence analysis data found a higher mutation rate in Sofosbuvir-treated cells, inferring an additional anti-ZIKV effect where sofosbuvir initiates mutations on the ZIKV genetic profile, causing elevated A–G mutation levels and increasing ZIKV susceptibility to replication error (Sacramento et al., 2017).

Recently, a novel RdRp inhibitor Favipiravir (6-fluoro-3-hydroxy-2-pyrazinecarboxamide or T-705) has been shown to promote neuronal cell growth and ameliorate apoptosis (Kim et al., 2018). It upregulates the AKT phosphorylation in the phosphatidylinositol-3-kinase (PI3K)/AKT pathway essential for neurogenesis and increases the expression of anti-apoptotic mediator Bcl-2, whereas pro-apoptotic factor Bax is reduced (Kim et al., 2018). ZIKV E and NS5 gene expression levels were also significantly reduced in hNPCs at 1, 10, and 25 μM, suggesting an effective suppression of ZIKV infection (Kim et al., 2018). Though the mode of interaction has yet to be elucidated, probable hypotheses are either direct misincorporation into the developing viral RNA sequence or indirect inhibition of transcription *via* attachment to polymerase (Baz and Boivin, 2019).

#### Others

A study using a mice model revealed an engineered AH peptide with D-enantiomer (AH-D) confers protection against ZIKV by stimulating viral liposome rupture (Jackman et al., 2018). Therapeutic administration of AH-D is reported to be able to abrogate ZIKV-related infection, apoptosis, and replication in neuronal and other systemic cells (Jackman et al., 2018). This finding is corroborated by a decrease in mortality and morbidity even when AH-D is given prophylactically (Jackman et al., 2018). Additionally, it can infiltrate the intact BBB without altering BBB permeability to reduce ZIKV burden and neuroinflammation in CNS through reduction of proinflammatory mediators, thus preventing BBB injury and neurodegeneration (Jackman et al., 2018).

## Conclusion

Following the sudden increase in the number of new-borns with microcephaly during the Brazillian outbreak of ZIKV, numerous studies have been conducted and they verify the causal relationship between ZIKV infection and neurological disorders. ZIKV possesses the ability to cross the placental barrier and BBB, targeting brain cells, particularly in the developing brain. The virus crosses the BBB through (i) transcellular pathway, (ii) monocyte transmigration through Trojan horse pathway, (iii) vascular leakage enhancement, and (iv) disruption of the choroid plexus epithelial layer. Upon crossing the BBB and entering the brain, ZIKV infects various cells in the developing brain, causing neuropathogenesis through (i) cell cycle perturbation, (ii) mitochondrial dysfunction, (iii) ER stress and UTP, (iv) modulation of host autophagy, and (v) neuronal apoptosis. Despite major advances in this field, many important questions remain answered: (i) Why only a small number of fetuses born to infected mothers develop microcephaly? What are the long-term outcomes of infected neonates without detectable abnormalities at birth? (ii) Is an intact BBB permissive to ZIKV infection? Can ZIKV target astrocytes in the adult brain? Will there be any long-term effects in adults infected with ZIKV? and (iii) Since the innate immune responses and mitochondria functions are connected, how does mitochondrial dysfunction upon ZIKV infection affect the cellular immune responses? Therefore, more studies are required to fully understand the mechanisms of cross-talk between ZIKV and the human brain, as well as the host factors involved *in utero* transmission and neuropathogenesis. In addition, systematic and long-term follow-ups are imperative to determine the unknown long-term neuropathological and behavioral consequences in both new-borns and adults.

Importantly, despite its severe implications and potential future outbreaks, there are still no vaccine or antiviral drugs available against ZIKV. The development of new therapeutic approaches should be one of the priorities for future research. The drug-repurposing strategy offers a promising avenue to identify potential antiviral drugs against ZIKV within a shorter development timeline and known safety. This approach is also particularly appealing for mosquito-borne viruses, which receive less attention from the affluent regions and pharmaceutical companies. A number of host-targeting and virus-targeting agents with potential inhibitory activity against ZIKV have been identified. However, the majority of these drugs have only been tested *in vitro*. It is also critical to evaluate their efficacy in appropriate animal models to better predict their clinical outcome. In addition, the ability of these drugs to prevent ZIKV CNS invasion needs to be evaluated. Importantly, ZIKV drugs should be clinically safe for use in pregnant women and fetuses.

## Author Contributions

LT and VB: conceptualization. LT and TK: methodology and writing—original draft preparation. LT, TK, WJ, and VB: writing, review, and editing. VB and WJ: supervision. All authors contributed to the article and approved the submitted version.

## Conflict of Interest

The authors declare that the research was conducted in the absence of any commercial or financial relationships that could be construed as a potential conflict of interest.

## Publisher’s Note

All claims expressed in this article are solely those of the authors and do not necessarily represent those of their affiliated organizations, or those of the publisher, the editors and the reviewers. Any product that may be evaluated in this article, or claim that may be made by its manufacturer, is not guaranteed or endorsed by the publisher.
